# Is *Taenia crassiceps* Cysticercosis a Threat to Dogs? Description of Macro- and Microscopic Lesions in a Dog. Case Report and a Review of the Literature

**DOI:** 10.3390/pathogens15010025

**Published:** 2025-12-24

**Authors:** Małgorzata Kandefer-Gola, Kacper Żebrowski, Rafał Ciaputa, Marta Demkowska-Kutrzepa, Stanisław Dzimira

**Affiliations:** 1 Department of Pathology, Faculty of Veterinary Medicine, Wroclaw University of Environmental and Life Sciences, Norwid Street 31, 50-375 Wroclaw, Poland; malgorzata.kandefer-gola@upwr.edu.pl (M.K.-G.); kacper.zebrowski@upwr.edu.pl (K.Ż.); rafal.ciaputa@upwr.edu.pl (R.C.); 2 Department of Parasitology and Fish Diseases, Faculty of Veterinary Medicine, University of Life Sciences in Lublin, Akademicka Street 12, 20-950 Lublin, Poland; marta.demkowska@up.edu.pl

**Keywords:** *Canis lupus familiaris*, cysticercosis, immunosuppression, prednisone, subcutaneous nodules, *Taenia crassiceps*, zoonosis

## Abstract

*Taenia crassiceps* is a cestode capable of causing severe and atypical cysticercosis in accidental intermediate hosts, including domestic dogs. Here we report a fatal disseminated *T. crassiceps* infection (cystiscercosis) in a 4-year-old castrated male German Shepherd from Poland that had been undergoing long-term prednisone therapy for Addison’s disease. The dog developed multiple soft subcutaneous nodules containing numerous asexually proliferating cysticerci. Necropsy revealed extensive dissemination of larvae throughout the subcutis and the thoracic and abdominal cavities, accompanied by serosanguineous effusions, necrosis, and chronic inflammatory lesions. Histological examination demonstrated cestode larvae with a scolex bearing two rows of hooks, consistent with *T. crassiceps*. Immunosuppression and endocrine alterations, including chronic glucocorticoid treatment and low levels of testosterone, likely promoted rapid asexual proliferation of larvae. A literature review shows that although dogs are definitive hosts of *T. crassiceps*, immunosuppressed individuals may also serve as accidental intermediate hosts. Early cytological evaluation of subcutaneous nodules may facilitate faster diagnosis and treatment decisions. Given the zoonotic potential of *T. crassiceps* and the increasing number of European cases, this parasitic infection should be considered in the differential diagnosis of subcutaneous nodules in immunosuppressed dogs. The presented case underscores its epidemiological relevance within the One Health framework.

## 1. Introduction

The tapeworm (*Taenia crassiceps*) (Zeder, 1800) is a member of the family Taeniidae with a complex life cycle, whose definitive hosts include foxes, dogs, and other carnivorous mammals [1,2]. The intermediate hosts are predominantly rodents and lagomorphs, in which the oncosphere migrates from the intestine to the subcutaneous tissues and body cavities, where it develops into the larval stage, formerly called *Cysticercus longicollis*. Following ingestion of the intermediate host by the definitive host, the larva enters the intestine, the scolex attaches to the mucosa, and the strobila begins to proliferate. The prepatent period ranges from 4 to 6 weeks [3].

In dogs, infection with *Taenia crassiceps* larvae may lead to cysticercosis involving subcutaneous tissues, musculature, body cavities, and internal organs. The larvae can multiply asexually by budding, facilitating rapid development of severe, disseminated infections [1,4]. Adult parasites typically inhabit the small intestines of foxes and dogs, and less commonly of cats. *T. crassiceps* infection has been documented in wild mammals across Europe and North America [5,6,7,8,9], as well as in zoological collections—for example, in the northern fur seal [10]. In domestic animals, cases of subcutaneous cysticercosis have been reported in the USA and several European countries, including Germany, Switzerland, and France [2,11,12,13,14]. In Poland, *T. crassiceps* has been identified in a ring-tailed lemur kept in a zoo, and in dog feces using polymerase chain reaction (PCR) diagnostics [15,16]. The first fatal case of subcutaneous cysticercosis in a domestic dog was described only recently [17]. Atypically presenting infections have also been reported in humans, both in immunocompromised and immunocompetent individuals [18,19]. In such circumstances, humans act as accidental intermediate hosts, and the larvae may develop in unusual anatomical locations, such as the subcutis or the central nervous system.

The present study aims to describe an autopsy-confirmed case of generalized cysticercosis caused by *T. crassiceps* larvae in a German Shepherd dog, and to review the most recent literature on this parasite.

## 2. Materials and Methods

A 4-year-old castrated male German Shepherd dog (*Canis lupus familiaris*), which had not left the territory of Poland, was submitted for necropsy. According to information provided by the owner, the dog had been treated with prednisone at a dose of 0.2 mg/kg b.w. twice daily (Prednicortone, Dechra Regulatory B.V., Bladel, the Netherlands) due to Addison’s disease. During therapy, numerous soft, nodular subcutaneous lesions appeared in various locations: the presternal region, right axillary area, right side of the neck, both inguinal regions, the perianal area, and the left hip and thigh extending to the hock. Surgical procedures were performed at these sites, including subcutaneous drainage and removal of necrotic debris and fluid containing numerous cysticerci. Draining tubes were also placed to allow exudate to drain. Despite symptomatic and causal treatment with praziquantel at a dose of 5.7 mg/kg b.w. (Anipracit, aniMedica GmbH, Senden-Bosensell, Germany), the dog died.

### 2.1. Necropsy and Sampling

A complete necropsy was performed according to standard veterinary pathology procedures. The dog was examined externally and internally, with particular attention to the condition of the skin, subcutaneous tissues, body cavities, and internal organs.

### 2.2. Cytology

Swabs were taken from wounds, subcutaneous tissue, and muscles, and fluid from the abdominal cavity was collected for cytological examination.

### 2.3. Histopathology

For histopathological examination, sections of the skin, subcutaneous tissue, and muscles from different areas of the dog’s body, and also internal organs, were collected, fixed in 10% buffered formalin, then paraffin blocks were made, and paraffin sections of 4 μm thick were cut. All slides were stained with haematoxylin and eosin (HE).

### 2.4. Parasite Identification

Identification of the cestode larvae was based on macroscopic, cytological, and histopathological examination. Species identification was performed using standard morphological keys and descriptions by Rommel et al. and Bowman, with particular emphasis on hook arrangement and morphology typical of *Taenia crassiceps* larvae (*Cysticercus longicollis*) [3,4].

All microscopic analysis was performed using an Olympus BX53 light microscope with an Olympus UC90 camera, using cellSens Standard V.1 software (Olympus, Tokyo, Japan).

## 3. Results

### 3.1. Gross (Necropsy) Findings

**Figure 1:**
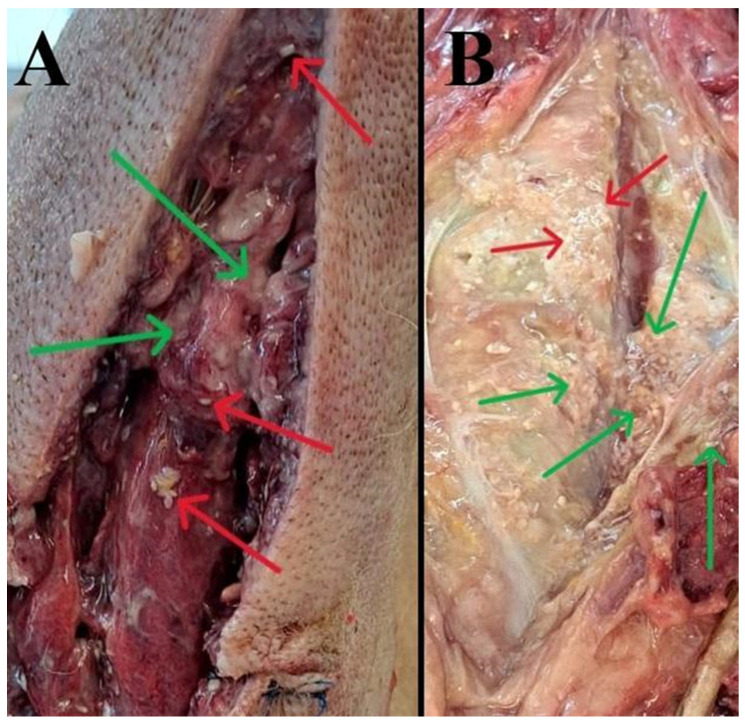
Numerous white structures measuring approximately 2–5 mm in length–tapeworm larvae located in the subcutaneous tissue (**A**) around the drains, which were placed intravitally. Numerous tapeworm larvae are found in fat and muscle tissue (**B**). Red arrows—tapeworm larvae, green arrows—necrotically damaged tissues.

Necropsy revealed edema, seropurulent inflammatory exudate, congestion, and focal necrosis of deeper tissues within the subcutaneous tissue and around the surgical wounds. Numerous 2–5 mm white cysticerci were present in parts of the subcutaneous tissue distant from the drains (Figure 1A,B). Marked edema and dystrophic mineralization (calcification) of the subcutis were observed in the perineal and scrotal regions.

**Figure 2:**
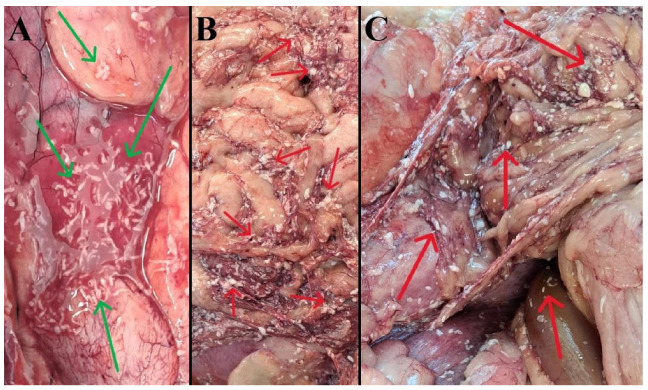
Tapeworm larvae (arrows) are approximately 5 mm in length on the surface of the thoracic cavity organs (**A**), on the surface of the omentum (**B**), and on the abdominal organs (**C**). Red and green arrows.

The thoracic cavity contained 100–150 mL of bloody fluid. Numerous tapeworm larvae were visible on the organs’ surfaces (Figure 2A). The lungs were intensely congested, with focal emphysema; the cut surfaces exhibited a slightly rough texture. The abdominal cavity contained approximately 500 mL of mild blood-tinged fluid. The omentum was covered with exudate and numerous larvae measuring approximately 5 mm (Figure 2B), similar to the surfaces of other organs (Figure 2C). The abdominal organs were in their normal anatomical position. The liver was light brown, the gallbladder was filled with liquid bile, and the bile ducts were patent. The stomach was mildly distended and contained about 100 mL of fluid content; the mucosa showed catarrhal inflammation and congestion. The contents of the small intestine were semi-fluid, and those of the large intestine were thicker. Numerous unformed fecal masses were present in the rectum. No adult tapeworms were found in the intestinal lumen.

### 3.2. Cytological Findings

**Figure 3:**
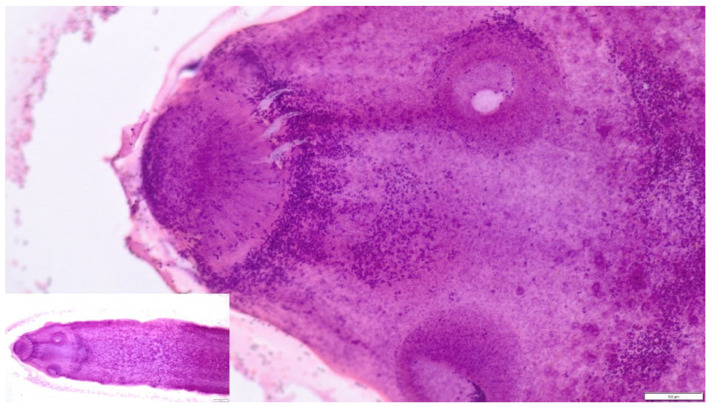
Larva from the fluid collected from the dog’s abdominal cavity, with visible suckers and partially preserved hooks. Hematoxylin and eosin (HE) stain, 100× magnification, inset 40×.

Microscopic examination of the whitish structures collected from the subcutaneous tissue and abdominal cavity revealed the presence of scolices with two rows of hooks (Figure 3). Numerous larval stages of tapeworms were present in the cavity fluid.

### 3.3. Histopathological Findings

**Figure 4:**
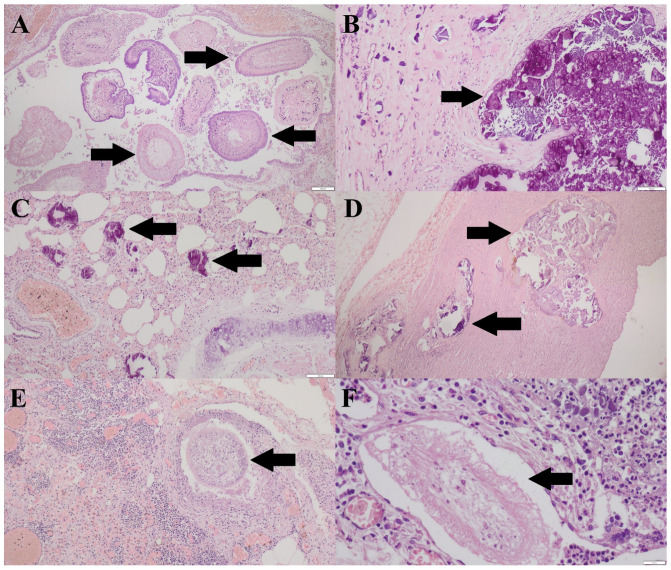
(**A**)—Numerous tapeworms (arrows) are visible within the parietal peritoneum. HE, 40×. (**B**)—Extensive dystrophic mineralization (calcification) (arrows) within the subcutaneous tissue. HE, 100×. (**C**)—Lungs with multiple foci of dystrophic calcification (arrows), emphysema, and congestion. HE, 100×. (**D**)—Aortic wall with distinct areas of dystrophic calcification (arrows). HE, 40×. (**E**)—Tapeworm present in the lymph node (arrow), accompanied by neutrophilic infiltration. HE, 100×. (**F**)—Peritoneal abscess containing a tapeworm larva (arrow). HE, 400×.

In skin, subcutaneous and muscle sections, multifocal, massive, chronic granulomas with numerous tapeworms were visible (Figure 4A). Additionally, dystrophic calcification occurred (Figure 4B). In the lung sections, diffuse dystrophic calcification with mild inflammatory reaction, followed by areas of edema, congestion, emphysema and atelectasis, was diagnosed (Figure 4C). In the aorta, multifocal calcification was disclosed (Figure 4D). In lymph nodes (both subcutaneous and internal ones), edema, inflammation (with occurrence of neutrophils and macrophages), and single tapeworms were visible (Figure 4E). In the omental and peritoneal sections, purulent inflammation with tapeworm larvae and single dystrophic calcifications were observed (Figure 4F). In other organs, only degenerative changes were found.

### 3.4. Parasitological Findings

Macroscopically, numerous whitish, oval larval structures measuring approximately 2–5 mm were observed within the subcutaneous tissues and body cavities. Cytological examination of aspirates and cavity fluids revealed scolices equipped with four suckers and a rostellum bearing two rows of hooks. The hooks had an arching claw (like a blade) that was markedly longer than the base, a morphological feature that is distinctive of *T. crassiceps.* Microscopic examination of isolated cysticerci led to a preliminary diagnosis of the species as *T. crassiceps.* Histopathological sections confirmed the presence of cestode larvae with a well-developed scolex and characteristic rostellar hooks.

## 4. Discussion

*Taenia crassiceps* is a cosmopolitan tapeworm widely distributed throughout the Northern Hemisphere and frequently detected in both wild and domestic canids [4,5,6,7,8,9,10]. Domestic dogs and wild canids such as red foxes (*Vulpes vulpes*) and wolves (*Canis lupus*) serve as the primary definitive hosts. By shedding eggs, they contribute to environmental contamination and maintenance of the parasite’s life cycle [3,5,6,9]. Notably, the literature emphasizes the high susceptibility of ring-tailed lemurs (*Lemur catta*) to *T. crassiceps* cysticercosis. Numerous European zoological gardens have reported cases of subcutaneous and disseminated infections in this species [20,21,22,23]. In some instances, larvae were also found within body cavities, suggesting a possible immunological dysfunction in these primates [24,25,26].

The relationship between the host’s immune response and endocrine status is of particular interest. In the early phase of infections, a Th1-dominated response prevails, whereas chronic infections favor a Th2 response that facilitates rapid larval proliferation. This mechanism has been described in detail in mice—larvae proliferate more rapidly in females than in males, and these differences disappear after castration, when testosterone levels decline, and estradiol concentration increases up to 100-fold [27]. Immune dysfunction plays a key role in humans as well. The most severe cases occur in individuals with human immunodeficiency virus (HIV) or other immunological impairments [18,28]. However, infections also occur in immunocompetent individuals, including cases of neurocysticercosis presenting with stroke-like symptoms [19]. It is worth noting that much of the available information on *T. crassiceps* is derived from studies on *Echinococcus multilocularis*, in which *T. crassiceps* is often detected as an accompanying species [14,16].

In the present case, the dog served as an atypical intermediate host, and larvae, most likely, *T. crassiceps* proliferated in the subcutaneous tissue, body cavities, and internal organs. Similar cases have been reported in the USA, Germany, France, and the Czech Republic [2,11,12,13,29,30]. The association between infection and endocrine disorders or immunosuppression appears particularly relevant. An example is the dog with Cushing’s syndrome described by Nolte et al. [13]. In the case presented here, prolonged prednisone therapy and castration—eliminating the protective role of testosterone—may have favored a shift toward a Th2-dominated response and rapid larval multiplication. This mechanism, well-documented in mouse models, thus appears to be reflected in dogs as well.

*Taenia crassiceps* is characterized by asexual larval proliferation via budding, which facilitates the development of massive infections [2,4,15]. Morphological examination of the larvae observed in the present case revealed features very consistent with *Taenia crassiceps* cysticerci, as described in the literature. The larvae measured approximately 2–5 mm in length and contained a single scolex with four suckers and a rostellum armed with two rows of hooks. Although precise morphometric measurements of individual hooks were limited by partial preservation, the general hook morphology was characteristic, with a distinctly elongated blade relative to the handle, a feature considered typical of *T. crassiceps* [3,4]. Comparable hook morphology and larval dimensions have been reported in dogs, lemurs, and humans affected by *T. crassiceps* cysticercosis [2,11,12,13,20,21,22,23].

It should be noted that some overlap in hook size exists among *Taenia* species, which may complicate species-level differentiation based solely on morphology. Nevertheless, the combination of hook arrangement, scolex structure, larval size, anatomical distribution, and the characteristic asexual budding pattern strongly supports the identification of *T. crassiceps* in the present case. Similar diagnostic approaches have been successfully applied in previously published veterinary and human case reports [2,13,18,30]. However, as the hook morphological features are not necessarily unique to *T. crassiceps*, additional testing, such as PCR, may be helpful in a more definitive identification [2]. Although PCR-based methods may provide definitive species identification, the diagnosis in this case was based on a combination of characteristic larval morphology, scolex and hook structure, typical anatomical distribution, and the clinical–pathological presentation, which together are considered sufficient for the identification of *Taenia crassiceps* as reported in previous veterinary and human case studies [2,13,18,30]. However, the lack of molecular confirmation of the parasite species is a limitation of this study.

Diagnosis is based on histopathological, microscopic, or molecular (PCR) examination [2,18,30]. Differential diagnosis should include other tapeworm species and neoplastic processes. Cytological examination of aspirates from subcutaneous lesions can significantly accelerate diagnosis. In clinical practice, cytology is often the first diagnostic step when proliferative lesions are suspected, but it may also reveal a parasitic etiology [31,32,33,34].

Treatment options include surgical removal of cysts and the use of albendazole, fenbendazole, or praziquantel; however, prognosis is poor in cases of disseminated infections. Experimental studies suggest the potential efficacy of combination therapy (albendazole with ivermectin) [35]. Environmental conditions favoring the persistence of *Taenia* spp. eggs—exceptionally moderate temperatures and high humidity—complicate control [36]. Various animal models used in cysticercosis research have played a crucial role in understanding tapeworm biology [37]. *T. crassiceps* itself constitutes an important experimental model, as its larvae can be maintained in inbred mice for prolonged periods, enabling the study of immunological and endocrine responses [38].

Infections with *T. crassiceps* can lead to severe and even fatal disease, not only in dogs. The rapid progression of illness and the larvae’s ability to undergo intensive asexual proliferation create a serious threat, particularly in hosts with impaired immunity, including individuals treated with glucocorticoids or those with immunosuppression. Therefore, *T. crassiceps* infections should be considered in the differential diagnosis of cutaneous and subcutaneous nodular lesions in dogs, especially in individuals with endocrine disorders or undergoing immunosuppressive therapy.

In the context of the increasing number of atypical and rare parasitic infections in Europe, both surveillance and documentation of such cases are of great importance.

Given its zoonotic potential and increasing occurrence in Europe, *T. crassiceps* cysticercosis should be considered not only a veterinary concern but also a relevant issue within the One Health framework.

## Data Availability

The datasets used and/or analyzed during the current study are available from the corresponding author upon reasonable request.

## Abbreviations

The following abbreviations are used in this manuscript:

PCR	polymerase chain reaction
HE	hematoxylin and eosin
HIV	human immunodeficiency virus
